# Molecular Characterization of Low-Density Polyethene (LDPE) Degrading Bacteria and Fungi from Dandora Dumpsite, Nairobi, Kenya

**DOI:** 10.1155/2018/4167845

**Published:** 2018-12-03

**Authors:** Christabel Ndahebwa Muhonja, Gabriel Magoma, Mabel Imbuga, Huxley Mae Makonde

**Affiliations:** ^1^Pan African University of Science and Technology, P.O. Box 62000-00200, Nairobi, Kenya; ^2^Jomo Kenyatta University of Agriculture and Technology, P. O. Box 62000-00200, Nairobi, Kenya; ^3^Department of Pure & Applied Sciences, Technical University of Mombasa, P. O. Box 90420-80100, Mombasa, Kenya

## Abstract

This study aimed at molecular and biochemical characterization of low-density polyethene (LDPE) degrading fungi and bacteria from Dandora dumpsite, Nairobi. Twenty bacterial and 10 fungal isolates were identified using 16S rDNA and 18S rDNA sequences for bacteria and fungi, respectively. The highest fungal degradation was attributed to *Aspergillus oryzae* strain A5,1 while the highest bacterial degradation was attributed to *Bacillus cereus* strain A5,a and *Brevibacillus borstelensis* strain B2,2, respectively. Isolates were screened for their ability to produce extracellular laccase and esterase; *Aspergillus fumigatus* strain B2,2 exhibited the highest presence of laccase (15.67 mm) while *Aspergillus oryzae* strain A5,1 exhibited the highest presence of esterase (14.33 mm). Alkane hydroxylase-encoding genes were screened for using primer AlkB 1 which amplified the fragment of size 870 bp. Four bacterial samples were positive for the gene. Optimum growth temperature of the fungal isolates was 30°C. The possession of laccase, esterase, and alkane hydroxylase activities is suggested as key molecular basis for LDPE degrading capacity. Knowledge of optimum growth conditions will serve to better utilize microbes in the bioremediation of LDPE. The application of *Aspergillus oryzae* strain A5,1 and *Bacillus cereus* strain A5,a in polyethene degradation is a promising option in this kind of bioremediation as they exhibited significantly high levels of biodegradation. Further investigation of more alkane degrading genes in biodegrading microbes will inform the choice of the right microbial consortia for bioaugmentation strategies.

## 1. Introduction

Low-density polyethene is a major cause of environmental pollution due to its high tensile strength, lightness, resistance to water, and microbial attack. The consumption of plastics in the country has increased to 4,000 tons per annum of polyethene bags which together with hard plastics end up scattered in the environment creating “the plastics menace” [1]. Through the National Environmental Management Authority (NEMA), Kenya has embraced the 3Rs, reduce, reuse, and recycle, concept of solid waste management [2] and most recently the ban on the use of polyethene carrier bags but this has not addressed the problem of polyethene which remains scattered in the environment [3].

Biodegradation is the decomposition of substances through microbial activity and is a complex process which involves the following steps [4]: biodeterioration, depolymerization, assimilation, and mineralization. Bacteria and fungi of various genera have been implicated previously in the biodegradation of polyethene albeit the low rates. *Acinetobacter sp.* was found capable of utilizing *n*-alkanes of chain length C10–C40 as a sole source of carbon as reported by [5]. Bacterial genera, namely, *Pseudomonas*, *Acinetobacter*, *Brevibacillus*, *Rhodococcus*, *and Micrococcus* [6, 7, 1], respectively, isolated from different sources proved to be the potential organisms for polyethene degradation. Fungal genera, *Gliocladium*, *Cunninghamella*, *Penicillium*, *Aspergillus*, *Fusarium*, *Mucor*, and *Mortierella*, from soil [1] were proven to have the potential to degrade polyethene after analysis of degradation through various methods.

Plastic biodegradation as a result of the activity of certain enzymes causes cleavage of the polymer chains into monomers and oligomers. Enzymatically broken down plastic is further absorbed by the microbial cell to be metabolized. Aerobic breakdown produces carbon dioxide and water. The involvement of enzymes in microbial biodegradation of polyethene has been investigated, and enzymes such as laccases and esterases have been confirmed to play a role in this process either directly or indirectly [8]. The production of enzyme laccase in the presence of polyethene as the sole carbon source is a clear indication that laccase has a role in breaking down some of the intermediary products produced during this process. In this study, molecular characterization of bacteria and fungi that had been confirmed to degrade polyethene was done as well as assessment of optimum pH, temperature, and sodium chloride concentration at which they can thrive. Presence of AlkB genes that encode alkane hydroxylases known to hydrolyze alkanes was also investigated.

## 2. Materials and Methods

### 2.1. Bacterial DNA Extraction

Total genomic DNA was isolated from the bacterial pure cultures grown to the late exponential phase by means of a standard protocol [9] as follows: 1.5 ml of the overnight bacterial culture (grown in the LB medium) was transferred to a 1.5 ml Eppendorf tube and centrifuged at 13000 rpm for 1 min to pellet the cells. The supernatant was discarded. The cell pellet was suspended in 600 *μ*l TE buffer and centrifuged at 13000 rpm and the supernatant discarded. The cell pellet was resuspended in 200 *μ*l TE buffer, and the following were added: 5 *μ*l lysozyme (20 mg/ml), 5 *μ*l RNAnase (20 mg/ml), and 10 *μ*l proteinase K (20 mg/ml) followed by overnight incubation at 37°C. In the next morning, the temperature was adjusted to 56°C for one hr and an equal volume of phenol/chloroform (1 : 1) was added and mixed well by inverting the tube until the phases were completely mixed. Spinning was done at 13000 rpm for 15 min at room temperature. The upper aqueous phase was carefully transferred to a new tube by using 1 ml pipette. This step was repeated twice to ensure all protein had been removed. An equal volume of chloroform and isoamyl (24 : 1) was added to the aqueous layer and centrifuged at 13000 rpm for 15 min. The aqueous layer was removed into a new tube. This step was also repeated to ensure all phenol is removed. An equal volume of isopropanol was added and stored overnight at −20°C. The samples were then defrosted and centrifuged at 4°C for 30 min to pellet the DNA. The pellet was washed in 70% ethanol and centrifuged at 13000 rpm for 5 min, and then, the ethanol was carefully pipetted out. The pellet was air dried on the bench for 20 min, and the isolated genomic DNA was viewed on a 1% agarose gel.

### 2.2. Bacterial DNA Amplification and Sequencing

Amplification of the 5′ end of the 16S rDNA gene was performed with universal primers (forward primer (8-F)5′-AGAGTTTGATYMTGGCTCAG-3′) and reverse primer ((1942R)5′-GGTTACCTTGTTACGACTT-3*'*) [10]. The PCR was performed on a GeneAmp PCR system 9600 (Applied Biosystems), using 1 *µ*l Taq Polymerase (Applied Biosystems), 1 *µ*l each of 10 pM concentrations of forward and reverse primers, 27 *µ*l sterile deionized water, 8 *µ*l PCR buffer containing dNTPs and MgCl_2_, and 2 *µ*l DNA template, for a total reaction volume of 40 *µ*l. The cycling program used was as follows: 1 cycle of 94°C for 5 min; 30 cycles of 94°C for 30 sec, 55°C for 30 sec, and 72°C for 1.5 min; and a final extension of 72°C for 10 min. The PCR products were visualized through electrophoresis on a 1% agarose gel with ethidium bromide added directly. The 1.5 kbp products were subjected to Sanger dideoxy sequencing using the forward primer and reverse primers at Macrogen DNA, Inc. (Netherlands). Sequence files were edited using Chromas version 2.6.2 and compared to the GenBank nucleotide database using the basic local alignment search tool (BLAST). Phylogenetic relationships were inferred using Mega 7 [11], and maximum-likelihood algorithms were available in Phylip. Maximum likelihood and parsimony-derived trees were bootstrapped using PHYML [12, 13].

### 2.3. Fungal DNA Extraction

Fungal DNA extraction protocol reported by Gontia-mishra et al. [14] was used. Fungal mycelia were grown for 7 days at 55°C on potato dextrose agar. Mycelia were frozen in liquid nitrogen and ground to a fine powder using a mortar and pestle. The powder was transferred into 2 ml tubes, and 600 *µ*l of preheated extraction buffer was added. The contents were incubated in a water bath at 65°C for 30 minutes with mixing after every 10 minutes. 270 *µ*l volume of 5 M potassium acetate was added and centrifuged at 13000 rpm for 10 minutes. 700 *µ*L of the supernatant was transferred into clean tubes, and 5 *µ*L RNASE (10 mg) was added and then incubated for 30 minutes at 37°C. Chloroform and iso-amyl alcohol was prepared in the ratio of 24 : 1, and an equal volume was added to the mixture. 600 *µ*L of supernatant was pipetted into clean tubes. DNA was precipitated by adding a tenth of the volume of 3 M potassium acetate and two thirds of the volume of isopropanol. This was incubated at −20°C for 30 minutes then centrifuged at 13000 rpm for 10 minutes. The pellet was washed using 70% ethanol followed by 10 minutes of centrifuging, and then, the DNA was eluted in 50 *µ*L of RNASE-free water and stored at −20°C.

### 2.4. Fungal DNA Amplification and Sequencing

PCR was performed on a GeneAmp PCR system 9600 (Applied Biosystems), using 1 *µ*l Taq Polymerase (Applied Biosystems), 1 *µ*l each of 10 pM of forward and reverse primers, 27 *µ*l deionized water, 8 *µ*l PCR buffer containing dNTPs and MgCl_2_, and 2 *µ*l DNA template, for a total reaction volume of 40 *µ*l. The cycling program used was as follows: 1 cycle of 95°C for 5 min; 35 cycles of 95°C for 30 sec, 60°C for 45 sec, and 72°C for 40 sec; and a final extension of 72°C for 5 min. Primer pair **F-**566:5′-CAGCAGCCGCGGTAATTCC-3′ and for R- 1200:5′-CCCGTGTTGAGTCAAATTAAGC-3′ which amplify on average a 650 bp long fragment from the V4 and V5 regions were used [15]. The PCR products were visualized through electrophoresis on a 1% agarose gel with ethidium bromide added directly. The products were subjected to Sanger dideoxy sequencing by Macrogen, Inc. (Netherlands). SeqMan Pro was used to assemble both the forward and reverse sequence files [16]. The sequences obtained were compared against the sequences available in the NCBI database using the basic local alignment tool (BLAST). The 18S rDNA gene sequences obtained in current study, together with those of the closest neighbor strains, were aligned using ClustaX version 2.1. Phylogenetic relationships were inferred using Mega 7 [11], and maximum-likelihood algorithms were available in Phylip. Maximum likelihood and parsimony-derived trees were bootstrapped using PHYML [12, 13].

### 2.5. Screening for Production of Enzymes

Bacterial isolates were screened for their ability to produce extracellular enzymes, i.e., laccases and esterases. The ability of the isolates to utilize substrates such as lignin and tween 20 exhibited their ability to produce the respective enzymes [17].

#### 2.5.1. Determination of Presence of Enzyme Laccase

The media for selection of lignin-modifying fungi were prepared by the use of plain agar and minimal salt media with the incorporation of lignin (to encourage selection of lygninolytic fungi) and Guaiacol, which acts as a colorimetric indicator of the lignin-modifying enzymes laccase or peroxidases. All chemicals were obtained from Sigma Chemical Co., St. Louis. The presence of a reddish coloration after 3–5 days of incubation was an indication of laccase activity. The laccase assay per 1 liter: 400 *μ*l Guaiacol, agar 15 g, 2 g malt extract, 0.5 g KH_2_PO_4_, 0.001 g ZnSO_4_, 0.4 g K_2_HPO_4_, 0.02 g FeSO_4_, and 0.2 g MgSO_4_, 0.5 g KH_2_PO_4_, 0.1 g NH_4_NO_3_, 0.1 g KCl, 5 ml KOH, 0.25 g chloramphenicol, forming a reddish colored zone as a positive result.

#### 2.5.2. Determination of Presence of Enzyme Esterase

The isolates were cultured on basal media (1% KH_2_PO_4_, 0.01% MgSO_4_·7H_2_O, 0.005% CaCl_2_·2H_2_O, 4% NaCl, and 1% Na_2_CO_3_) supplemented with 1% tween 20 (domestic grade) as the sole carbon source. The medium was then thereafter inoculated by the spotting of isolates per plate and incubated for at least 48 hours at 37°C for bacteria and at 28°C for 3–5 days for fungal isolates. The media were observed for zones of precipitation of calcium crystals around each isolate. Positive isolates for esterases production were indicated by the precipitation of calcium crystals around the colonies.

#### 2.5.3. Screening for Genes Producing Alkane-Degrading Enzymes

Amplification was done using the sets of AlkB primers [18] shown in Table 1. The PCR was performed on a GeneAmp PCR system 9600 (Applied Biosystems) using Taq DNA polymerase. A total of 30 cycles of amplification was performed with template DNA denaturation at 94°C for 1 min, primer annealing at 40°C for 1 min, and primer extension at 72°C for 2 min [19]. The PCR products were visualized through electrophoresis on a 1% agarose gel with 2 *µ*l ethidium bromide added directly.

### 2.6. Effect of Temperature on Growth of Fungal Isolates

Potato dextrose agar augmented with 250 mg/ml ampicillin to inhibit bacterial growth at pH 7.0 was prepared, sterilized, and dispensed in sterile Petri dishes. Each plate was inoculated with one fungal isolate and incubated at temperatures 20, 30, and 40^o^C. Growth of isolates was checked after 4 days of incubation. The level of growth was scored using the colony diameter, whereby 0 mm indicated no growth, 1-2 mm indicated minimal growth, 3-4 mm indicated average growth, and 5–7 mm indicated satisfactory growth while 8–10 mm indicated excellent growth.

## 3. Results and Discussion

### 3.1. Phylogenetic Relatedness of Low-Density Polyethene Degrading Fungal Isolates

**Figure 1:**
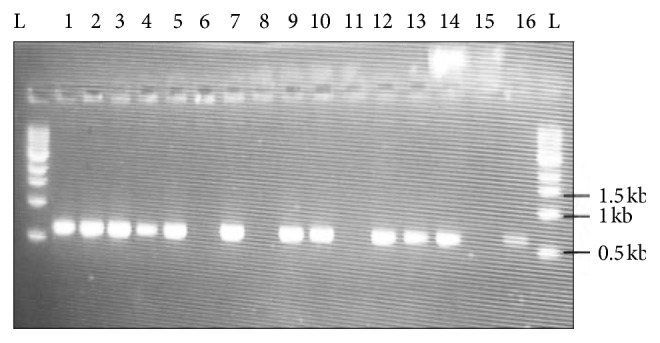
PCR products for the amplification of 18S rDNA for the fungal isolates 1, 2, 3, 4, 5, 6, 7, 8, 9, 10, 11, 12, 13, 14, 15, and 16 using 1200R and 566F universal primers. L represents a 1 kb ladder. The expected band size amplified is 640 bps.

**Figure 2:**
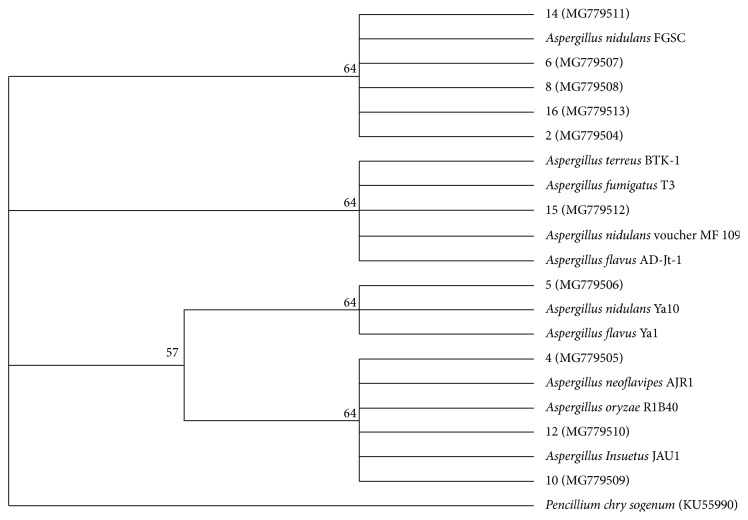
Phylogenetic tree of fungal isolates based on 18S rDNA sequences. All screened fungal isolates have NCBI accession codes in brackets. The scale bar refers to 0.007 substitutions per nucleotide position. Bootstrap values obtained with 1000 resampling are referred to as percentages at all branches.

Amplification of fungal 18S rDNA using 1200R and 566F universal primers yielded the expected band size of approximately 640 bps from the PCR products of the amplified samples (Figure 1). These products were purified, sequenced, and analyzed. The results were used to obtain accession numbers from the NCBI GenBank. The analyzed sequences were aligned with those of the closest neighbors using ClustalX version 2.1. Phylogenetic relationships were inferred from phylogenetic comparison of the 18S rDNA sequences using Mega 7 and maximum-likelihood algorithms to generate the phylogenetic tree (Figure 2) which shows the phylogenetic relationships among the various *Aspergillus* species. The tree displays four clades in which the isolates have been clustered. From our previous study [20], *Aspergillus oryzae* (MG779508) resulted in a weight loss of 36.4 ± 5.53% which was the highest. *Aspergillus oryzae* is a promising biodegrader of polyethene as it was able to degrade 30% of polyethene in 200 days [21] in addition to formation of microcracks and increased embrittlement of the LDPE surface upon SEM analysis. In a study done using untreated LDPE incubated with *A. oryzae*, 5% weight loss was recorded compared with control (untreated and unexposed). *Aspergillus fumigatus* strain B2,2 (MG779513) recorded a weight reduction of 24 ± 3.26% which was the second highest in our previous study [20]. *Aspergillus fumigatus* is also among the species that have been investigated for their ability to degrade polyethene and other polymers. In a study, three fungal species were investigated for their ability to degrade polyethene, and *A. fumigatus* was the best degrader compared to *A. terreus* and *F. solani* following an analysis of the LDPE surface by SEM and FTIR [22]. Other fungi implicated in this study included *Aspergillus nidulans*, *A. flavus*, *A. terreus*, and *A. neoflavipes* which resulted in weight loss of the LDPE sheets. Use of weight reduction as a measure of the extent of polyethene biodegradation has been widely accepted and used by many authors [23].

### 3.2. Phylogenetic Relatedness of Low-Density Polyethene Degrading Bacterial Isolates

**Figure 3:**
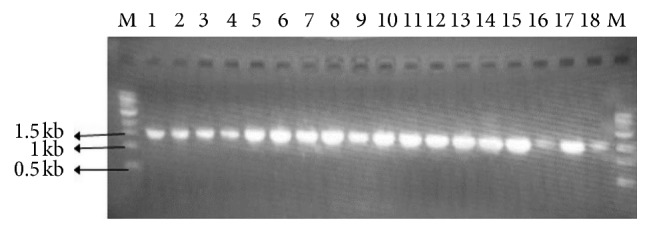
Amplification of 16S rDNA for the bacterial isolates 1–18 using 1492R and 8F universal primers. M represents a 1 kb marker. The expected band size amplified is 1420 bps.

**Figure 4:**
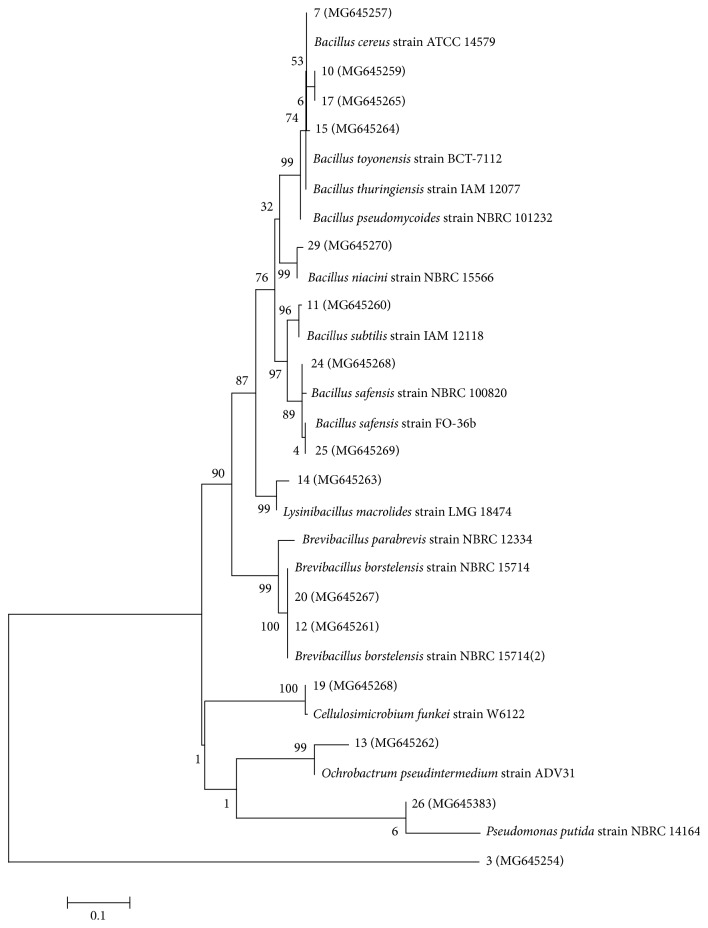
Phylogenetic tree generated by MEGA 7 for 16S rDNA sequences of the bacterial isolates found to be effective bacterial degraders. All screened bacterial isolates have NCBI accession codes in brackets. The scale bar refers to 0.02 substitutions per nucleotide position. Bootstrap values obtained with 1000 resamplings are referred to as percentages at all branches.

Amplification of bacterial 16S rDNA using 1492R and 8F universal primers yielded 1420 bps fragments (Figure 3) which were purified, sequenced, and analyzed. The results were used to obtain accession numbers from the NCBI GenBank. The analyzed sequences were aligned with those of the closest neighbors using ClustaX version 2.1. Phylogenetic relationships were inferred from phylogenetic comparison of the 16S rDNA sequences using Mega 7 and maximum-likelihood algorithms to generate the phylogenetic tree (Figure 4) which shows the phylogenetic relationships among the genera and species. *Brevibacillus*, *Bacillus*, and *Lysinibacillus* are in one major clade while *Pseudomonas*, *Ochrobactrum*, and *Cellulosimicrobium* are grouped in another major clade. Bacteria of the genera *Bacillus*, *Brevibacillus*, *Ochrobactrum*, *Lysinibacillus*, *Cellulosimicrobium*, and *Pseudomonas* were identified as effective polyethene degraders. Bacterial isolates A5,1a-*Bacillus cereus* (MG645256) produced the highest degradation effectiveness in terms of weight loss, i.e., 35.2%, followed by the isolate B2,2-*Brevibacillus borstelensis* (MG645267) showing 20.28% from an earlier study [20] while isolates B1,1a-*Pseudomonas putida* (MG645283) and D4,yn-*Brevibacillus borstelensis* strain (MG645261) produced a weight loss of 2.88% and −6.8%, respectively. The genus *Bacillus* was most frequently identified among the LDPE biodegrading genera in this study. Species identified under this genus include *Bacillus cereus*, *Bacillus toyonensis*, *Bacillus thuringiensis*, *Bacillus subtilis*, *Bacillus pseudomycoides*, *Bacillus safensis*, and *Bacillus niacini.* Various studies have been done to investigate the efficacy of genus *Bacillus* in polyethene degradation, and different species under this genus have been found to have potential to degrade polyethene [24, 25]. *Bacillus cereus* has been found to be a good bioremediation candidate in the biodegradation of polyethene due to its ability to produce enzymes laccase and manganese peroxidase. In a comparative study, *B. cereus* was found to be more effective than *B. sphericus* in degrading photo-oxidized and thermos-oxidized LDPE [26]. According to [6], *Brevibacillus borstelensis* Accession number AY764129 was able to degrade 11% of nonirradiated polyethene by weight in 30 days. Two bacterial isolates *Bacillus amyloliquefaciens* (BSM-1) and *Bacillus amyloliquefaciens* (BSM-2) were isolated from municipal soil and used for polymer degradation studies and were found to produce significant changes on LDPE in terms of weight loss, reduction of tensile strength, and appearance of new functional groups [27]. A novel strain of *Pseudomonas*, *Pseudomonas citronellolis* EMBS027, GenBank Accession number KF361478 was isolated by [28] from a municipal landfill in Indore, India, and it degraded 17.8% of polyethene in 4 days. Different species of *Pseudomonas* were analyzed for their ability to degrade polyethene and upon incubation for 120 days. *Pseudomonas putida* resulted in a weight loss of 9% [29].

### 3.3. Screening for Enzyme Production

**Figure 5:**
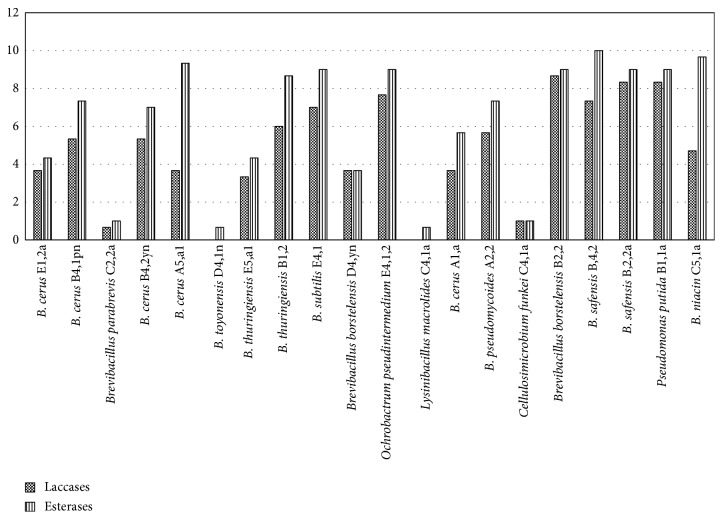
Presence of enzymes laccase and esterase among the bacterial isolates. Growth of bacterial isolates was measured as colony diameter (mm). Means were grouped using Tukey's honest significant difference test at *P* < 0.05.

**Figure 6:**
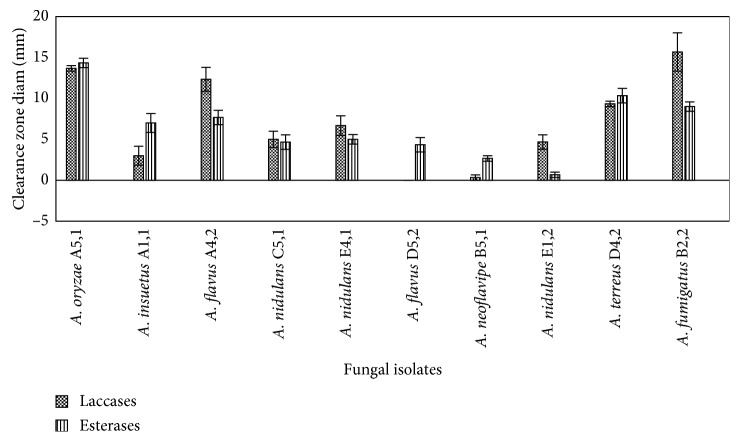
Presence of enzymes laccase and esterase among the fungal isolates. Growth of fungal isolates was measured as colony diameter (mm). Means were grouped using Tukey's honest significant difference test at *P* < 0.05.

Bacterial isolates were screened for production of enzymes laccase and esterase (Figure 5) which are among the enzymes implicated in LDPE degradation. Bacterial isolates *Brevibacillus borstelensis* strain B2,2, *Brevibacillus parabrevis* strain C2,2a, and *Pseudomonas putida* strain B1,1 exhibited the highest presence of laccase. Only two isolates *Bacillus toyonensis* and *Bacillus macrolides* were negative for laccase activity. Esterase activity was highest in isolates *Brevibacillus borstelensis* strain D4 yn, *Bacillus niacin* strain C4,1a, and *Pseudomonas putida* strain B1,1a. Fungal isolates were screened for production of enzymes laccase and esterase (Figure 6). Isolates B2, 2-*Aspergillus fumigatus*, A5,1-*Aspergillus oryzae*, and A4,2a-*Aspergillus flavus* exhibited the highest levels of laccase enzyme while the highest level of esterase enzyme was attributed to fungi *Aspergillus Oryzae*.

Production of extracellular enzymes plays an important role in polymer degradation through depolymerization, where the polymer is broken down into smaller subunits [30] which are then enzymatically degraded into intermediary products that can be assimilated into microbial cells [31] and utilized as carbon sources leading to production of energy, water, carbon dioxide, and methane in the case of anaerobic respiration [32]. In this study, production of extracellular enzymes, laccase and esterase, were investigated. Fungal and bacterial isolates in this study were scrutinized for their ability to produce laccase enzyme and isolate B2, 2-*Aspergillus fumigatus* (MG779513) which had a LDPE degradation effectiveness of 24% and had the highest diameter of coloration due to laccase production. This could be attributed to its ability to produce higher amounts of laccase and other extracellular enzymes which are believed to play a role in polyethene degradation. According to [33], the production of this enzyme increases when the microbes are in close proximity with the polyethene. Reference [34] was able to extract the crude laccase enzyme which was incubated with polyethene and led to degradation as was evidenced through weight loss, FTIR, and SEM. Esterases catalyze the cleavage of ester bonds [35] of short-chain triglycerides or esters. Esters have been identified as part of the intermediary products produced during polyethene degradation when the postincubation culture media are subjected to GC-MS analysis that can be assimilated into the microbial cells, undergoes hydrolysis to give rise to the subsequent carboxylic acid and alcohol that ultimately undergo respiration to produce energy [36]. Isolate A5,1-*Aspergillus oryzae* (MG779508) with a weight loss of 36.4% had a high activity of enzyme esterase of 10%. This could have contributed to its high degradation potential compared to other fungal isolates which had lower degradation.

### 3.4. Screening for AlkB Genes Producing Alkane-Degrading Enzymes

**Figure 7:**
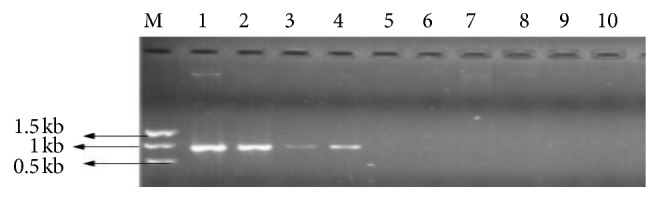
PCR products for the amplification of Alkb for the bacterial isolates 1, 2, 3, 4, 5, 6, 7, 8, 9, and 10 using AlkB 1 set 1 primers. Lane 1 represents a 1 kb ladder. The expected band size amplified is 870 bps.

PCR to amplify AlkB genes was done using three sets of AlkB primers. Only one set of the AlkB primer was able to amplify the AlkB gene producing a fragment of size 870 bps. AlkB genes are responsible for production hydrolase enzymes which are responsible for alkane degradation. The gene was amplified in 4 bacterial samples (Figure 7). A common feature of many alkane degraders is that they contain multiple alkane hydroxylases with overlapping substrate ranges [37]. AlkB- and alkB-related genes code for an alkane-degrading enzyme, alkane hydroxylase [38]. The analysis of the bacterial samples revealed presence of AlkB 1 gene in 4 of the bacterial samples. Bacterial isolates that were positive for alkB 1 gene were D4 yn-*Brevibacillus borstelensis*, B1,1-*Pseudomonas putida*, and A5,a1- *Bacillus cereus.* Alkane biodegradation is initiated through terminal oxidation to the corresponding primary alcohol, which is further oxidized by dehydrogenases to fatty acids which can enter the TCA cycle [39]. This genetic information is an indication of the genetic ability of the microorganisms to degrade long-chain alkanes through production of this enzyme.

### 3.5. Effect of Temperature on Growth of Fungal Isolates

**Figure 8:**
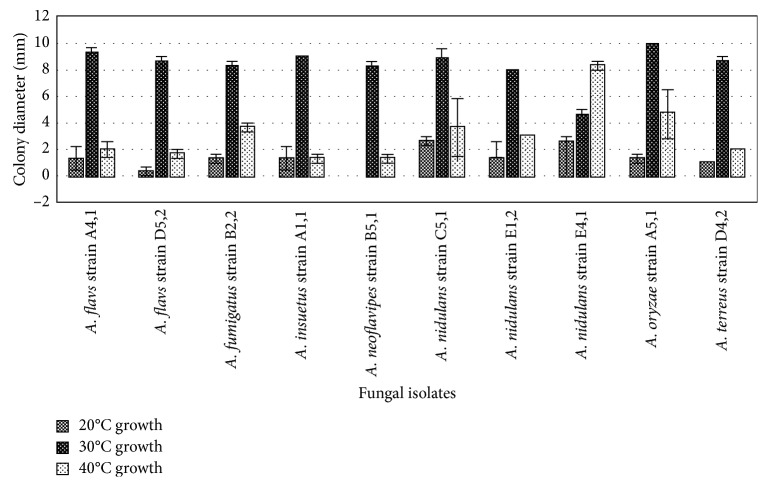
Growth of fungal isolates at 20°C, 30°C, and 40°C, respectively.

The growth of fungi at different temperatures (20°C, 30°C, and 40°C) as shown (Figure 8) revealed that growth at 30°C was significantly higher than growth at 20°C and 40°C with *A. oryzae* strain A5,1 having the highest growth (10 ± 0). However, isolate E4,1-*A. nidulans* grew optimally at 40°C. This could be attributed to the fact that the sampling site for these bacteria was from a dumpsite where the temperatures were generally ambient and hence favoring the growth of mesophilic microbes. Laccase production by fungi is influenced by type and concentration of carbon sources, pH, and temperature.

## 4. Conclusion

The present work indicates that soil bacteria and fungi isolated from the dumpsite have potential of degrading polyethene. This is the first study on the isolation of local bacteria and fungi that can degrade LDPE which is the most common plastic in Kenya. Particularly, the application of *Aspergillus oryzae* strain A5,1 and *Bacillus cereus* strain A5,a will be beneficial in the bioremediation of polyethene as they exhibited significant degradation effectiveness. It was ascertained that the microorganisms are capable of producing enzymes laccase and esterase which have been confirmed to play a role in degradation of polyethene. The isolates possess the alkane hydroxylase-producing gene (AlkB) which is the molecular explanation for the degradation of LDPE under investigation. Fungi in this study were found to grow optimally at the temperature of 30°C.

## Figures and Tables

**Table 1:** Primers for AlkB genes encoding depolymerases responsible for alkane degradation [18].

Primers and position	PCR product	Reference
*alkB 1 set 1*		
82 5′-TGGCCGGCTACTCCGATGATCGGAATCTGG-3′ 111	870 bp	Kok et al.
951 5′-CGCGTGGTGATCCGAGTGCCGCTGAAGGTG-3′ 922		

*alkB 1 set 2*		
134 5′-CATTTCCCTGGTGATTG-3′ 151	718 bp	Stover et al
851 5′-CCGTCCTCGCCCTTTCGC-3′ 834		

*alkB 2*		
134 5′-CCTGGCTGGTGATCAGCG-3′ 151	749 bp	Stover et al.
882 5′-CGAGTGTTCCGGCGTGGTG-3′ 864		

## Data Availability

The data (Figures 1–8 and Table 1) used to support the findings of this study are included within the article.
